# A review on the effect of macrocyclic lactones on dung-dwelling insects: Toxicity of macrocyclic lactones to dung beetles

**DOI:** 10.4102/ojvr.v82i1.858

**Published:** 2015-04-16

**Authors:** Carmen T. Jacobs, Clarke H. Scholtz

**Affiliations:** 1 Department of Zoology and Entomology, University of Pretoria, South Africa

## Abstract

Avermectins and milbemycins are commonly used in agro-ecosystems for the control of parasites in domestic livestock. As integral members of agro-ecosystems with importance in maintaining pasture health through dung burial behaviour, dung beetles are an excellent non-target bio-indicator taxon for examining potential detrimental effects of pesticide application. The current review focuses on the relative toxicity of four different anthelmintics (ivermectin, eprinomectin, doramectin and moxidectin) in dung residues using dung beetles as a bio-indicator species. One of the implications of this review is that there could be an effect that extends to the entire natural assemblage of insects inhabiting and feeding on the dung of cattle treated with avermectin or milbemycin products. Over time, reduced reproductive rate would result in decreased dung beetle populations and ultimately, a decrease in the rate of dung degradation and dung burial.

## Introduction

The importance placed on anthelmintics to bring parasite populations under control has resulted in a challenging arms race to develop a product that exhibits the perfect balance between host and non-target organism toxicity and pest resistance. The need for more effective products is becoming increasingly important because pest resistance appears to be keeping pace with the development of new products. Pest resistance is arguably one of the top challenges as far as protecting livestock is concerned and probably the main driving force behind parasite control research in the livestock industries (Sangster 1999; Wolstenholme *et al.* 2004) as it has been reported in many countries, in a variety of nematodes and against all currently available anthelmintics (Sutherland & Leathwick 2011).

Anthelmintics, which control helminth pests by removing them, are grouped according to their common chemistry and mode of action (Sangster & Dobson 2002; Vercruysse & Rew 2002). Currently, the avermectins (ivermectin, eprinomectin and doramectin) and the milbemycins (moxidectin), collectively known as macrocyclic lactones, are amongst the most effective anthelmintics on the market.

The avermectins are naturally produced by strains of a soil-dwelling actinomycete, *Streptomyces* (Burg *et al.* 1979; Shoop & Soll 2002). All the avermectins have a unique pharmacophore that consists of a 16-membered macrocyclic lactone backbone (Shoop & Soll 2002) with a disaccharide chain at C-13 (Steel 1993; Vercruysse & Rew 2002). Although the avermectins are a glycosidic derivative of the pentacyclic 16-membered lactone (Albers-Schoenberg *et al.* 1981; Chabala *et al.* 1980), they do not possess the antifungal and antibacterial properties associated with the macrolide antibiotics (Albers-Schoenberg *et al.* 1981; Burg *et al.* 1979; Chabala *et al.* 1980). They act by interfering with invertebrate neurotransmission rather than inhibiting protein synthesis (Albers-Schoenberg *et al.* 1981; Chabala *et al.* 1980).

Ivermectin was the first avermectin to be introduced in 1981 (Steel 1993; Vercruysse & Rew 2002). Ivermectin (22, 23-dihydroavermectin) is a disaccharide derivative of the pentacyclic 16-membered lactone (Burg *et al.* 1979; Campbell 1985; Chabala *et al.* 1980; Römbke *et al.* 2010). The antiparasitic effect of ivermectin is extremely potent against insects, nematodes and acarines (Campbell 1985; Putter *et al.* 1981). Although potent, ivermectin is not equally active against all species and is often highly stage specific (Campbell 1985), so that a genus known to be susceptible to ivermectin may not be susceptible at all life stages (Campbell & Benz 1984).

Abamectin, a combination of 80% avermectin B_1a_ and 20% avermectin B_1b_, is the starting material for ivermectin. It is effective against nematodes as well as acarines and to date remains the only avermectin or milbemycin to be used in both the animal health and crop industries (Shoop, Mrozik & Fisher 1995).

Eprinomectin was introduced to the animal health industry in 1997 as an alternative to ivermectin as it was considered to be the only topical endectocide safe for use in lactating dairy animals (Shoop *et al.* 1996b; Vercruysse & Rew 2002). Although ivermectin has no side-effects on the host and has a very broad spectrum of activity, with few exceptions it cannot be used in lactating dairy animals because of the levels of residue that remain in the milk (Shoop *et al.* 1996a, 1996b; Vercruysse & Rew 2002).

Doramectin was commercialised in 1993 (Vercruysse *et al.* 1993) and is the easiest avermectin to administer. In a study by Grandin, Maxwell and Lanier (1998), it was found that doramectin caused significantly less discomfort during administration than ivermectin.

The milbemycins, although structurally similar and with a similar range of biological activity to the avermectins, differ in substituents in a few of the side chains at the C-13 position and can basically be considered to be deglycosylated avermectins (Sangster & Dobson 2002; Steel 1993; Vercruysse & Rew 2002). Although they were discovered in 1973, before the discovery of ivermectin, they were originally developed for use in crop protection and have been used in veterinary practice from about 1986 only (McKellar & Benchaoui 1996; Takiguchi *et al.* 1980).

Moxidectin, the only milbemycin available on the market as an endectocide, was introduced in 1989 and commercialised worldwide by the early 1990s (McKellar & Benchaoui 1996; Steel 1993). The milbemycins are highly lipophilic (moxidectin is about 100 times more lipophilic than the avermectins), soluble in organic solvents and insoluble in water, and after an initial increase in its plasma concentration post-administration, it is redistributed throughout the body fat reserves, from which it is slowly released (McKellar & Benchaoui 1996).

Various studies have shown that a characteristic of the avermectins, regardless of the animal or method of administration, is that most of the dose is excreted largely unaltered in the dung, where it retains its insecticidal activity (Campbell 1985; Steel 1993; Strong 1993; Wardhaugh & Rodriguez-Menendez 1988). This is the focus of the present review.

## Published studies

Numerous laboratory and field studies have been undertaken on the effects of avermectins and milbemycin in cattle dung on non-target organisms and on their effects on different aspects of dung beetle biology. Countries with large cattle populations were chosen based on Food and Agriculture Organization of the United Nations, Statistic Division (FAOSTAT)'s live animal production database (Food and Agriculture Organization of the United Nations [FAO] 2013). Although the methods used were different in each country and changed somewhat over the years, the results have remained more or less consistent.

### Ivermectin

Ivermectin is the most extensively studied of all the avermectins. The first study that set the scene for interest in the field was that of Wall and Strong (1987), who conducted an experiment in the UK to investigate the environmental consequences of treating cattle with ivermectin. In contrast to the control dung pats, the experimental pats contained few to no Coleoptera or Diptera. The results also indicated that there was no visible dung degradation in the ivermectin-treated dung when compared to the controls. This field trial showed that treatment with a ruminal bolus that delivers 40 µg/kg ivermectin per day was enough to disrupt the entire dung-inhabiting insect community. Various subsequent studies have simulated or repeated this experiment with variable results.

#### Lethal and sublethal effect studies

Lumaret *et al.* (1993) studied the effects of ivermectin residues on dung beetles by running a field trial on a farm in Spain in spring. Dung toxicity was assessed by recording the mortality of the dung beetles feeding on the dung. In addition, the numbers of larvae and pupae were recorded after 29 days. No adult mortality was recorded for the duration of the study but 100% larval and pupal mortality was observed in dung collected on the day after treatment. No differences in offspring numbers between treated and untreated dung were observed from day 6 onwards. A delay in development was observed for beetles bred in treated dung when compared to the control offspring. Pitfall traps baited with dung collected 10 and 17 days after treatment were similarly attractive with treated and untreated dung for the first 3 days, and then a peak of attraction occurred between days 4 and 6, when the dung was most attractive and still relatively fresh. From day 6 onwards, the attraction to the treated dung persisted for 30 days whilst the untreated dung became unattractive after day 7. Lumaret *et al.* (1993) proposed that increased attractiveness is a result of biochemical modifications in the dung composition, most likely as a result of protein degradation released by ivermectin therapy.

Krüger and Scholtz (1997) ran a laboratory trial to determine the lethal and sublethal effects of ivermectin residues in dung from animals treated with a single standard injection of ivermectin at 200 µg/kg. Laboratory colonies of *Euoniticellus intermedius* were provided with 250 mL of dung twice a week for 2 weeks and monitored for adult mortality as well as for brood ball numbers. Brood balls were counted, removed and incubated to monitor for emergence. No results regarding adult survival were reported. There was no significant difference between treated and control populations in the number of brood balls formed; however, on average, the number of adults emerging from treated brood balls was significantly lower than in the controls (similar findings were obtained by Fincher [1992]). Ivermectin caused 100% mortality in offspring 2–7 days after treatment and significantly fewer emergences from day 14 after treatment when compared to the controls. Prolonged development in treated broods (similar to the findings of Lumaret *et al.* [1993]) was also recorded, roughly 2.5 times longer for dung collected 1, 7 and 14 days after treatment and a larval developmental time of 5 weeks compared to the control of 3.5 weeks for dung collected 28 days after treatment.

#### Survival and reproduction studies

Ridsdill-Smith (1988) studied the effect of ivermectin on the survival and reproduction of the dung beetle *Onthophagus binodis* in Australia. Ivermectin had no influence on adult dung beetle survival. Immature survival, however, was zero for week 1 after treatment but steadily rose to equal that of the other anthelmintic by week 8 after treatment. There was no untreated control.

Fincher (1992) compared the effect of 20 µg/kg and 200 µg/kg ivermectin on some dung-inhabiting insects, including the introduced African dung beetle *E. intermedius* in Texas, USA. The results revealed that neither dosage had any significant effect on adult survival, as described by Ridsdill-Smith (1988) and Wardhaugh and Rodriguez-Menendez (1988), or brood ball production when compared to the controls; however, emergence of adult *E. intermedius* from brood balls made with dung from cattle that received 200 µg/kg ivermectin was reduced for no more than 2 weeks after treatment (Fincher 1992).

Cruz Rosales *et al.* (2012) evaluated the effect of ivermectin on the survival and fecundity of *E. intermedius* adults as well as on the survival and development of *E. intermedius* from egg to adult in Mexico. They found that at low concentrations (10 µg/kg) the ivermectin had no effect on the survival or fertility of the adults or on the survival of the larvae, but they did record an increase in the larval development time. At the medium concentration (1 mg/kg) the survival of adults was reduced to almost half and no larvae emerged. At the highest concentration (100 mg/kg) 100% mortality was observed and no oviposition was performed. They concluded that the prolonged development time may cause a phase lag in the field activity cycle, which may reduce the number of *E. intermedius* individuals and the efficiency of the environmental services that they provide, and that more analyses with higher concentrations between 0.01 ppm and 0.1 ppm of ivermectin are needed to establish lethal concentrations for larvae and adults of *E. intermedius*.

#### Dung decomposition studies

Wardhaugh and Rodriguez-Menendez (1988) studied the effect of ivermectin on the development and survival of the dung beetles *Copris hispanus*,* Bubas bubalus* and *Onitis belial* in southern Spain. The results showed no adult mortality, reduced egg-laying and reduced juvenile survival as described by Ridsdill-Smith (1988). A marked reduction in adult feeding activity was observed in treatments suffering the highest mortalities, namely day 1–8 dung, and the inference was made that mortality was a result of the accumulating toxic effects, which suppressed feeding (Wardhaugh & Rodriguez-Menendez 1988). Whilst this study was aimed at the development and survival of the dung beetles, a decrease in the rate of dung decomposition as a result of reduction in adult feeding activity was observed.

Madsen *et al.* (1990) conducted field as well as laboratory experiments in Denmark to show how treating cattle with a single therapeutic ivermectin injection affected the fauna and decomposition of dung pats. The results from the field trial showed that ivermectin had an effect on beetle larvae 1–10 days after treatment but that the number of larvae was not affected by ivermectin applied 20–30 days before collection. The decomposition rate was significantly delayed when compared to control dung but also depended on variables such as climate, season, soil type, faunal inhabitants and microclimate. The results from the laboratory bioassays showed a 95% – 100% mortality rate in *Musca domestica* as well as *Musca autumnalis* for dung collected one day after treatment. There was no clear reduction in excreted ivermectin placed in the field for 7–62 days and the 62-day assay was obscured by natural mortality. Most of the variance found in this experiment was attributed to seasonal conditions.

Sommer *et al.* (1992) ran a field trial in Denmark to assess the impact of ivermectin residues on dung fauna and the resulting effect on dung degradation. According to the arthropods found in the treated dung, there was no significant difference between the residues found in the pour-on and injectable formulations even though the pour-on formulation was 2.5 times the dose of the injectable formulation; however, dung collected from cattle 1–2 days after treatment with the injectable formulation showed delayed dung degradation for up to 45 days but no effect was observed on dung collected 13–14 days after treatment. Dung collected from cattle 1–2 days after treatment with the pour-on formulation led to delayed dung degradation for up to 13–14 days after treatment, which was a similar result to that of Wardhaugh and Rodriguez-Menendez (1988) and Madsen *et al.* (1990).

Iglesias *et al.* (2011) evaluated the local effects of ivermectin on dung fauna and degradation under different meteorological and biological conditions in the same area in Argentina in 2011. The results showed that fewer arthropods were found in the dung of the calves treated with ivermectin, but the difference was not statistically significant.

#### Community structure studies

Krüger and Scholtz (1998a, 1998b) conducted a large-scale field study to determine the ecotoxicological effect of ivermectin on the dung beetle community structure under drought and high rainfall conditions. The results showed a large effect on the dung beetle community in the form of significantly lower species richness and evenness as well as increased species dominance in treated dung during drought conditions (Krüger & Scholtz 1998a). During high rainfall conditions, however, fewer beetle and fly larvae were found in the pats after 7 days, but no effect of ivermectin was detected after a year (Krüger & Scholtz 1998b). This suggests that these ecotoxicological effects are likely to be more severe in times of drought than under more favourable conditions.

Kryger, Deschodt and Scholtz (2005) carried out a long-term, large-scale field study in South Africa to assess the effect of ivermectin on the structure of dung beetle communities. No observable effects of ivermectin on the dung beetle communities were found, as the disparities between treated and untreated dung were insignificant and most probably a result of differences in microclimate. Species richness and diversity were also unaffected and ecologically similar to the control communities. This study showed that treatment with ivermectin under extensive farming conditions in the South African Highveld can be considered safe with regard to the dung beetle communities under high rainfall conditions.

Strong *et al.* (1996) carried out a comparative field trial to examine the effects of ivermectin and fenbendazole boluses on dung-colonising Diptera and Coleoptera in the UK. Although there were no significant differences in adult beetle numbers between the treated and untreated dung, not only was there a significant difference in larval and pupal numbers between the ivermectin and fenbendazole treated and untreated dung, but the larvae found in the ivermectin-treated dung were inhibited in their development. Pitfall trapping showed no significant difference in adult beetle numbers between treated and untreated dung, although a trend towards higher numbers of beetles attracted to the treated dung was noted.

Römbke *et al.* (2010) carried out a field study in Spain to determine the effects of ivermectin on the structure and function of dung and soil invertebrate communities. They observed a significantly lower abundance of adult dung beetles on the dung from cattle treated with ivermectin compared to the control group. They also noted that although adult dung beetles were attracted to the ivermectin-spiked dung, the rate of degradation was slower than for the control dung.

#### Dung attractiveness studies

Errouissi and Lumaret (2010) studied the effects on the attractiveness to dung beetles of dung treated with ivermectin. They found that the ivermectin-contaminated dung showed a significant attractive effect, which highlighted the danger of wide-spread ivermectin use as this potentially puts the dung beetles’ offspring and, indirectly, future beetle generations’ survival at risk.

### Eprinomectin and doramectin

Only comparative studies involving the effect of these products on dung beetles were available and are discussed in the next section, but two studies involving effects on other taxa are briefly described.

Lumaret *et al.* (2005) examined the larvicidal activity of eprinomectin residues on the dung-inhabiting fly *Neomyia cornicina* in France and found that eprinomectin residues in dung had a significant effect on *N. cornicina* as no emergences were observed on the dung from days 1–11 but after day 12 the first flies emerged.

Floate *et al.* (2008) addressed concerns raised about the use of endectocides affecting birds that feed on dung-breeding insects by testing the toxicity of faecal residues after doramectin treatment. A significant reduction in insect emergence was noted for dung from cattle treated ≤ 4 weeks prior, which was attributed to higher concentrations of the residues.

### Moxidectin

Fincher and Wang (1992) tested the effects of moxidectin on two introduced African species of dung beetle, namely *E. intermedius* and *Onthophagus gazella*. They found no significant differences between the mean number of brood balls produced by either species or on the emergence of progeny between treated and untreated dung. There also seemed to be no effect on the sex ratio for either species. They concluded that moxidectin seemed to be compatible with beneficial dung-burying beetles when used at the recommended dose.

Iwasa, Suzuki and Maruyama (2008) examined the effects of moxidectin on non-target coprophilous insects, more specifically the dung beetle *Caccobius jessoensis*, in cattle dung in field as well as laboratory trials in Japan. The results showed that concentrations were at maximum levels 3 days after treatment, showed a marked decline by day 7 and were not detectable by day 21. No significant differences were found between the control and the treated cattle dung with regard to numbers and weight of brood balls as well as emergence rates. Results of the field study, again, showed no significant differences between the control and the treated cattle dung. They concluded that moxidectin has no, or at most, the least effect compared to other avermectins on non-target coprophagous insects.

### Comparative studies: Comparison of two products

Comparative studies have been undertaken between ivermectin and doramectin (Dadour 2000; Suárez *et al.* 2003; Webb *et al.* 2010); ivermectin and moxidectin (Doherty *et al.* 1994; Strong & Wall 1994); moxidectin and doramectin (Suárez *et al.* 2009) and moxidectin and eprinomectin (Wardhaugh, Longstaff & Morton 2001).

Dadour (2000) examined the impact that abamectin and doramectin have on the survival and reproduction of the dung beetle *O. binodis*. This study was carried out in Australia and abamectin, rather than ivermectin, was chosen because it was the first avermectin sold commercially for the treatment of endoparasites in Australia. Significant adult mortality was observed in abamectin-treated dung 3–6 days after treatment and in doramectin-treated dung 9 days after treatment. Whereas abamectin residues had no effect on adult mortality in sexually mature beetles, sexually immature (newly emerged) beetles, which went through a period of intense feeding during which they were exposed to maximum abamectin residues, were found to be much more affected by the residues. In contrast to other studies (Fincher 1992; Krüger & Scholtz 1997), brood ball production was also significantly lower in beetles fed on dung from cattle treated with abamectin for up to 42 days after treatment. Brood ball production was also significantly lower in beetles fed on dung from cattle treated with doramectin, but only for 3–6 days after treatment. The enhanced brood mass in beetles fed on dung from doramectin-treated cattle at 24–34 days after treatment could not be explained. According to the high-performance liquid chromatography (HPLC) results, doramectin reached maximum concentration on day 3 after treatment, following a linear decline, with an elimination half-life of 15 days (Dadour 2000).

Suárez *et al.* (2003) compared the effects of ivermectin and doramectin on the invertebrate colonisation of cattle dung in Argentina. No significant differences were found in the numbers of adult beetles, regardless of the treatment. Faecal residue concentrations for both ivermectin and doramectin were highest in the first few days and remained relatively high throughout the experimental period. Doramectin concentrations were higher than ivermectin concentrations, as the results showed that after 180 days of exposure to environmental conditions, dung collected 27 days after ivermectin treatment still contained 56% residue compared to dung collected from doramectin treatment, which contained 75% residue.

Webb *et al.* (2010) assessed the abundance and dispersal of dung beetles in response to ivermectin and doramectin treatment on pastured cattle in Scotland by running a 2-year field trial. In the field-scale study, significantly more beetles were trapped in fields grazed by cattle treated with an avermectin than in fields where cattle remained untreated. The colonising trials, however, indicated that *Aphodius* beetles preferred colonising dung from untreated cattle rather than dung from cattle treated with doramectin and could discriminate between dung from untreated cattle and dung from cattle treated with doramectin at a spatial scale of at least 70 m.

Doherty *et al.* (1994) compared the larvicidal activities of different concentrations of moxidectin and abamectin on *O. gazella* to assess the level of threat they pose to dung fauna, and consequently dung degradation, in Australia. Although oviposition was not affected by either treatment, larval survival was affected by all concentrations of abamectin and by all concentrations of moxidectin over 128 µg/kg. In fact, moxidectin at 256 µg/kg and 512 µg/kg produced survival comparable to 4 µg/kg and 8 µg/kg abamectin.

Strong and Wall (1994) compared the relative effects of ivermectin and moxidectin on the colonisation of dung by dung-inhabiting insects in England. There was no significant difference between the three treatments in adult Scarabaeidae numbers showing that neither ivermectin nor moxidectin residues repel colonising adult beetles. However, dung collected from ivermectin-treated cattle up to 7 days after treatment showed high larval mortality, unlike moxidectin-treated dung and the control.

Suárez *et al.* (2009) demonstrated the effects of moxidectin and doramectin faecal residues on the activity of dung-colonising insects by depositing dung from cattle treated with moxidectin, dung from cattle treated with doramectin and control dung from untreated cattle on a field. Comparisons of dung degradation were inconclusive; however, total numbers of insects recovered from control pats were significantly higher than in treated pats. Furthermore, a lower adverse effect was observed for moxidectin compared to doramectin with no significant degradation of moxidectin or doramectin observed.

Wardhaugh *et al.* (2001) compared eprinomectin to moxidectin by examining the survival and development of *Onthophagus taurus* when fed on dung from treated cattle in Australia. The results showed that moxidectin had no effect on the survival or development of the beetles but the opposite was found to be true for eprinomectin. High juvenile mortality and suppressed brood ball production amongst those that survived were recorded. They concluded by designing a model that simulated the effects of eprinomectin residues and suggested that a single treatment of eprinomectin is capable of reducing the next generation by 25% – 35%.

### Comparative studies: Comparison of all four products

Two laboratory studies provided comparative results amongst ivermectin, moxidectin, doramectin and eprinomectin but they were performed under different laboratory conditions (Floate 2007; Floate, Colwell & Fox 2002). Floate (2006) also wrote a review about the global environmental effects of faecal residues left by treatment of cattle with ivermectin, doramectin, moxidectin and eprinomectin on non-target dung-inhabiting species.

Pour-on formulations of ivermectin, doramectin, eprinomectin and moxidectin were applied to four groups of heifers in Canada at the recommended dose of 500 µg/kg and dung was collected 1, 2, 4 and 6 weeks after treatment. Artificial dung pats were then randomly deposited in a block design in a pasture adjacent to grazing cattle and collected again after 8 days to analyse insect populations. To monitor dung beetle activity, dung-baited pitfall traps were placed in the centre and at either end of the study site. Based on the number of species affected and duration of suppression, the results showed that treatment of cattle with doramectin, ivermectin, eprinomectin or moxidectin, in descending order of adverse effect and reduced levels of insect activity in the dung but moxidectin was the least likely to affect the natural insect assemblage associated with cattle dung (Floate *et al.* 2002).

Floate (2006) raised concerns that the use of endectocides in cattle may reduce the insect diversity in Canada and lead to the accumulation of undegraded dung on pastures as a result of reduced insect activity required for dung pat degradation.

Floate (2007) also compared the field effects of ivermectin, doramectin, eprinomectin and moxidectin residues on the attractiveness of dung to dung-colonising insects over 3 years in Canada. Pitfall traps were set in spring and autumn and re-baited weekly for a month in each season. Insect captures were compared between pitfall traps baited with dung from untreated cattle and dung from cattle treated with doramectin, eprinomectin, moxidectin or ivermectin at the recommended dose of 500 µg/kg. Twofold and up to sixfold differences in captures between control and treated dung were observed. More specifically, 11 out of 29 cases of attraction and 11 out of 29 cases of repellence were recorded for doramectin, eprinomectin tended to repel insects, with 19 out of 29 cases of repellence, whilst ivermectin (17 out of 25 cases) and moxidectin (17 out of 18 cases) showed a strong attractive effect. Floate (2007) concluded that emergence of offspring from field-colonised dung should not be used as a measure of residue toxicity; standardised laboratory tests should still be the preferred method, but rather as a measure of ‘insect activity’, which is a composite measure of residual toxicity, the number and species composition and the mortality factors such as predation, competition and parasitism.

## Effect of routes of administration on faecal concentration

There are a variety of ways to administer avermectins to cattle, namely subcutaneously by injection, topically in the form of a pour-on and orally in various forms. Lumaret *et al.* (2005) determined the faecal concentrations of pour-on eprinomectin in cattle following treatment at the recommended dose of 500 µg/kg by using HPLC. The maximum faecal concentrations were recorded 3 days after treatment. Eprinomectin remained detectable in the faeces until 29 days after treatment. Lumaret *et al.* (1993) measured ivermectin concentrations in dung from cattle treated with a single dose of injectable ivermectin at the recommended dose rate of 200 µg/kg by using HPLC. Chemical analysis of the ivermectin concentration in fresh dung indicated that it increased daily on days 1–4 after treatment, reaching a peak of elimination on day 5 followed by a rapid decrease until day 12, after which the concentration was under the detection limit.

One would expect that the injectable formulations would be more effective than the pour-on formulation but this is not always the case. In the Denmark field trial by Sommer *et al.* (1992), the concentration of subcutaneously administered ivermectin was compared to the pour-on formulation of ivermectin using HPLC. Although there was no significant difference between the residue concentrations of the pour-on and injectable formulations, even though the pour-on formulation was 2.5 times the dose of the injectable formulation, the injectable formulation led to a longer period of delayed dung degradation than the pour-on formulation.

Herd, Sams and Ashcraft (1996) examined the persistence of ivermectin in faeces by comparing the faecal residues following different modes of administration, namely sustained-release (SR) bolus, pour-on and injectable formulations, in Ohio, USA. They emphasised the importance of formulation and route of administration in drug concentration determination, persistence and ecotoxic potential. All faecal concentrations recorded, regardless of mode of administration, were well above concentrations that are lethal or sublethal to beneficial dung-breeding invertebrates. They concluded by stating that the SR bolus and pour-on formulations are likely to be more ecotoxic to non-target organisms than the injectable formulation judging from their higher faecal concentrations, and that the SR bolus formulation is of particular concern because of the persistent excretion of toxic concentrations for prolonged periods of time.

## The way forward

Most recently, Wall and Beynon (2012) wrote a review on the impact of macrocyclic lactone parasiticides. They reported that macrocyclic lactone residues from parasiticide treatments may play an important role in the loss of coprophilous insects, which may in turn delay pat decomposition. They added that field studies have provided contradicting results that reflect confounding factors such as weather conditions, pat moisture content, pat location, time of year, dung insect species phenologies, timing and method of application. These factors are important in determining whether the results obtained from experimental and laboratory studies reflect the real impact on the economically important process of dung decomposition. The timely removal of dung from pastures by insects and weathering is both functionally and economically important; if appropriate decomposition does not occur, cattle farmers may suffer considerable economic losses as a result of pasture fouling, increases in dung-breeding pest fly populations and a higher transmission of livestock endoparasites. The benefits of rapid dung removal are therefore rather substantial; not only does it reduce such losses, but it helps to return nutrients to the soil, particularly nitrogen, a large proportion of which would otherwise be lost as ammonia.

## Conclusion

Although it is difficult to recommend a control programme that will suit all forms and styles of livestock farming, a standardised procedure for the testing of antiparasitic remedies needs to be developed in order to accurately compare the toxicity of various products. The best scenario would be to farm holistically, minimising the need for pesticides.
